# A comparison and user-based evaluation of models of textual information structure in the context of cancer risk assessment

**DOI:** 10.1186/1471-2105-12-69

**Published:** 2011-03-08

**Authors:** Yufan Guo, Anna Korhonen, Maria Liakata, Ilona Silins, Johan Hogberg, Ulla Stenius

**Affiliations:** 1 Computer Laboratory, University of Cambridge, 15 JJ Thomson Avenue, Cambridge CB3 0FD, UK; 2 Institute of Environmental Medicine, Karolinska Institutet, SE-171 77, Stockholm, Sweden; 3 Department of Computer Science, Aberystwyth University, SY23 3DB, UK

## Abstract

**Background:**

Many practical tasks in biomedicine require accessing specific types of information in scientific literature; e.g. information about the results or conclusions of the study in question. Several schemes have been developed to characterize such information in scientific journal articles. For example, a simple section-based scheme assigns individual sentences in abstracts under sections such as Objective, Methods, Results and Conclusions. Some schemes of textual information structure have proved useful for biomedical text mining (BIO-TM) tasks (e.g. automatic summarization). However, user-centered evaluation in the context of real-life tasks has been lacking.

**Methods:**

We take three schemes of different type and granularity - those based on section names, Argumentative Zones (AZ) and Core Scientific Concepts (CoreSC) - and evaluate their usefulness for a real-life task which focuses on biomedical abstracts: Cancer Risk Assessment (CRA). We annotate a corpus of CRA abstracts according to each scheme, develop classifiers for automatic identification of the schemes in abstracts, and evaluate both the manual and automatic classifications directly as well as in the context of CRA.

**Results:**

Our results show that for each scheme, the majority of categories appear in abstracts, although two of the schemes (AZ and CoreSC) were developed originally for full journal articles. All the schemes can be identified in abstracts relatively reliably using machine learning. Moreover, when cancer risk assessors are presented with scheme annotated abstracts, they find relevant information significantly faster than when presented with unannotated abstracts, even when the annotations are produced using an automatic classifier. Interestingly, in this user-based evaluation the coarse-grained scheme based on section names proved nearly as useful for CRA as the finest-grained CoreSC scheme.

**Conclusions:**

We have shown that existing schemes aimed at capturing information structure of scientific documents can be applied to biomedical abstracts and can be identified in them automatically with an accuracy which is high enough to benefit a real-life task in biomedicine.

## Background

The past decade has seen great progress in the field of biomedical text mining (BIO-TM). This progress has been stimulated by the rapid publication rate in biosciences and the need to improve access to the growing body of textual information available via resources such as the National Library of Medicine's PubMed system [1]. In recent past, considerable work has been conducted in many areas of BIO-TM. Basic domain resources such as biomedical dictionaries, ontologies, and annotated corpora have grown increasingly sophisticated, and a variety of novel techniques have been proposed for the processing, extraction and mining of information from biomedical literature. Current systems range from those capable of named-entity recognition to those dealing with e.g. document classification, information extraction, segmentation, and summarization, among many others [2-6].

While much of the early research on BIO-TM concentrated on technical developments (i.e. adapting basic language processing techniques for biomedical language), in recent years, there has been an increasing interest in users' needs [7]. Studies exploring the TM needs of biomedical researchers have appeared [8-10], along with practical tools for the use of scientists [11-14]. However, user-centered studies are still lacking in many areas of research and further evaluation of existing technology in the context of real-life tasks is needed to determine which tools and techniques are actually useful [15].

In this article we will focus on one active area of BIO-TM research - *textual information structure *of scientific documents - and will investigate its practical usefulness for a real-life biomedical task. The interest in information structure (also called *discourse, rhetorical, argumentative *or *conceptual *structure, depending on the theory or framework in question) stems from the fact that scientific documents tend to be fairly similar in terms of how their information is structured. For example, many documents provide some background information before defining the precise objective of the study in question, and conclusions are typically preceded by a description of the results obtained. Many readers of scientific literature are interested in specific information in certain parts of documents, e.g. in the general background of the study, the methods used in the study, or the results obtained). Accordingly, many BIO-TM tasks have focused on the extraction of information from the relevant parts of documents only. Classification of documents according to the categories of information structure has proved useful e.g. for question-answering, summarization and information retrieval [16-18].

To date, a number of different schemes have been proposed for (typically) sentence-based classification of scientific literature according to categories of information structure, e.g. [16,19-25]. The simplest of these schemes merely classify sentences according to section names seen in scientific documents, for example, the Objective, Methods, Results and Conclusions sections appearing frequently (with different variations) in biomedical abstracts [20,21,24]. Some other schemes are based on components of scientific argumentation. A well-known example of such a scheme is the Argumentative Zoning (AZ) scheme originally developed by Teufel and Moens [16] which assumes that the act of writing a scientific paper corresponds to an attempt of claiming ownership for a new piece of knowledge. Including categories such as Other, Own, Basis and Contrast, AZ aims to model the argumentative or rhetorical process of convincing the reviewers that the knowledge claim of the document is valid.

Also schemes based on conceptual structure of documents exist - for example, the recent Core Scientific Concepts (CoreSC) scheme [25]. CoreSC treats scientific documents as humanly readable representations of scientific investigations. It seeks to retrieve the structure of an investigation from the paper in the form of generic high-level concepts such as Hypothesis, Model, and Experiment (among others). Furthermore, schemes aimed at classifying statements made in scientific literature along qualitative dimensions have been proposed. The multi-dimensional classification system of Shatkay et al. [23], developed for the needs of diverse users, classifies sentences (or other fragments of text) according to dimensions such as Focus, Polarity, Certainty, Evidence and Trend.

Different schemes of information structure have been evaluated in terms of inter-annotator agreement, i.e. the agreement with which two or several human judges label the same element of text with the same categories. Some of the schemes have been further evaluated in terms of machine learning: the accuracy with which an automatic classifier trained on human-annotated data is capable of assigning text to scheme categories, e.g. [16,21,24,26]. Also evaluation in the context of BIO-TM tasks such as question-answering, summarization, and information retrieval has been conducted [16-18]. These evaluations have produced promising results. However, evaluation in the context of real-life tasks in biomedicine has been lacking, although such evaluation would be important for determining the practical usefulness of the schemes for end-users.

In this paper, we will investigate the usefulness of information structure for Cancer Risk Assessment (CRA). Performed manually by human experts (e.g. toxicologists, biologists), this real-life task involves examining scientific evidence in biomedical literature (e.g. that available in the MEDLINE database [27]) to determine the relationship between exposure to a substance and the likelihood of developing cancer from that exposure [28]. The starting point of CRA is a large-scale literature review which focuses, at the first instance, on scientific abstracts published on the chemical in question. Risk assessors read these abstracts, looking for a variety of information in them, ranging from the overall aim of the study to specific methods, experimental details, results and conclusions [29]. This process can be extremely time consuming since thorough risk assessment requires considering all the published literature on a chemical in question. A well-studied chemical may well have tens of thousands of abstracts available (e.g. MEDLINE includes over 27,500 articles for cadmium). CRA is therefore an example of a task which might well benefit from annotations according to textual information structure.

Our study focuses on three different schemes: those based on section names, AZ and CoreSC, respectively. We examine the applicability of these schemes to biomedical abstracts used for CRA purposes. Since AZ and CoreSC have been developed for full journal articles, our study provides an idea of their applicability to tasks involving abstracts. We describe the annotation of a corpus of CRA abstracts according to the three schemes, and compare the resulting annotations in terms of inter-annotator agreement and the distribution and overlap of scheme categories. Our evaluation shows that for all the schemes, the majority of categories appear in scientific abstracts and can be identified by human annotators with good or moderate agreement (depending on the scheme in question). Interestingly, although the three schemes are based on entirely different principles, our comparison of annotations reveals a clear subsumption relation between them.

We introduce then a machine learning approach capable of automatically classifying sentences in the CRA corpus according to scheme categories. Our results show that all the schemes can be identified using automatic techniques, with the accuracy of 89%, 90% and 81% for section names, AZ and CoreSC, respectively. This is an encouraging result, particularly considering the fairly small size of the CRA corpus and the challenge it poses for automatic classification.

Finally, we introduce a user test - conducted by experts in CRA - which evaluates the usefulness of the different schemes for real-life CRA. This test focuses on two schemes: the coarse-grained scheme based on section names and the finest-grained CoreSC scheme. It evaluates whether risk assessors find relevant information in literature faster when presented with unannotated abstracts or abstracts annotated (manually or automatically) according to one of the schemes. The results of this test are promising: both schemes lead into significant savings in risk assessors' time. Although manually annotated abstracts yield biggest savings in time (16-46%, compared with the time it takes to locate information in unannotated abstracts), considerable savings are also obtained with automatically annotated abstracts (11-33% in time). Interestingly, although CoreSC helps to save more time than section names, the difference between the two schemes is so small that it is not statistically significant.

In sum, our work shows that existing schemes aimed at capturing information structure can be applied to biomedical abstracts relatively straightforwardly and identified automatically with an accuracy which is high enough to benefit a real-life task.

The rest of this paper is organized as follows: The Methods section introduces the CRA corpus, the annotation tool, and the annotation guidelines, together with the automatic classification methods and the methods of direct and user-based evaluation. The Results section describes first the annotated corpus. The results of the inter-annotator agreement tests, comparison of the schemes in annotated data, the automatic classification experiments, and the user-test are then reported. The Discussion and Conclusions section concludes the paper with comparison to related research and directions for future work.

## Methods

### The three schemes

Full journal articles are more complex and richer in information than abstracts [30]. As a distilled summary of key information in full articles, abstracts may exhibit an entirely different distribution of scheme categories than full articles. For the practical tasks involving abstracts, it would be useful to know which of the existing schemes are applicable to abstracts and which of them can be identified in them automatically with sufficient accuracy. We chose three different schemes for our investigation - those based on section names, Argumentative Zones, and Core Scientific Concepts:

• **Section Names - S1**: The first scheme differs from the other two in the sense that it is actually developed for abstracts. It is based on section names found in some scientific abstracts. We use the 4-way classification from [21] where abstracts are divided into Objective, Method, Results and Conclusions. Hirohata et al. show that this 4-way classification is the most frequently used classification in MEDLINE abstracts [27]. They also provide a mapping of the four section names to their synonymous names appearing in MEDLINE. Table 1 provides a short description of each category and its abbreviation (for this and for other schemes). For example, the Objective category (OBJ) of this scheme aims to capture the background and the aim of the research described in abstracts.

**Table 1:** The three schemes

S1	Objective	OBJ	The background and the aim of the research
	Method	METH	The way to achieve the goal
	Result	RES	The principle findings
	Conclusion	CON	Analysis, discussion and the main conclusions
**S2**	Background	BKG	The circumstances pertaining to the current work, situation, or its causes, history, etc.
	Objective	OBJ	A thing aimed at or sought, a target or goal
	Method	METH	A way of doing research, esp. according to a defined and regular plan; a special form of procedure or characteristic set of procedures employed in a field of study as a mode of investigation and inquiry
	Result	RES	The effect, consequence, issue or outcome of an experiment; the quantity, formula, etc. obtained by calculation
	Conclusion	CON	A judgment or statement arrived at by any reasoning process; an inference, deduction, induction; a proposition deduced by reasoning from other propositions; the result of a discussion, or examination of a question, final determination, decision, resolution, final arrangement or agreement
	Related work	REL	A comparison between the current work and the related work
	Future work	FUT	The work that needs to be done in the future

**S3**	Hypothesis	HYP	A statement that has not been yet confirmed rather than a factual statement
	Motivation	MOT	The reason for carrying out the investigation
	Background	BKG	Description of generally accepted background knowledge and previous work
	Goal	GOAL	The target state of the investigation where intended discoveries are made
	Object	OBJT	An entity which is a product or main theme of the investigation
	Experiment	EXP	Experiment details
	Model	MOD	A statement about a theoretical model or framework
	Method	METH	The means by which the authors seek to achieve a goal of the investigation
	Observation	OBS	The data/phenomena recorded within an investigation
	Result	RES	Factual statements about the outputs of an investigation
	Conclusion	CON	Statements inferred from observations and results, relating to research hypothesis

• **Argumentative Zoning - S2**: The second scheme is based on Argumentative Zoning (AZ) of documents. AZ provides an analysis of the rhetorical progression of the scientific argument. It follows the knowledge claims made by authors. Teufel and Moens [16] introduced AZ and applied it first to computational linguistics papers. Mizuta et al. [19] modified the scheme for biology papers. More recently, Teufel et al. [22] introduced a refined version of AZ and applied it to chemistry papers. As the recent refined version of AZ is too fine-grained for abstracts (many of the categories do not appear in abstracts at all) and is not directly applicable to biomedical texts (the annotation guidelines need to be supplemented with domain-specific terminology and rules for individual categories - see [22] for details), we adopt the earlier version of AZ developed for biology papers [19]. From the ten categories of Mizuta et al., we select seven which (according to our pilot investigation) actually appear in abstracts: those shown in Table 1. Note that we have re-named some of the original category names, mostly for improved annotation accuracy.

• **Core Scientific Concepts - S3**: The third scheme is the recent concept-driven and ontology-motivated scheme of Liakata et al. [25]. This scheme views papers as written representations of scientific investigations and aims to uncover the structure of the investigation as Core Scientific Concepts (CoreSC). Like AZ, CoreSC has been previously applied to chemistry papers [25,31]. The CoreSC is a 3-layer annotation scheme but we only consider the first layer in the current work. The second layer pertains to properties of the categories (e.g. "advantage" vs. "disadvantage" of METH, "new" vs. "old" METH). Such level of granularity is rare in abstracts. The 3rd layer involves co-reference identification between the same instances of each category, which is also not of concern in abstracts. We adopt the eleven categories in the first layer of CoreSC (shown in Table 1). S3 is thus the most fine-grained of our schemes.

### Data: cancer risk assessment abstracts

We used as our data the corpus of CRA abstracts described in [29]. It contains MEDLINE abstracts from 15 biomedical journals (e.g. *Carcinogenesis, Chemico-biological Interaction, Environmental Health Perspectives, Mutation Research, Toxicological Sciences*) which are used frequently for CRA purposes and which jointly provide a good coverage of the main types of scientific evidence relevant for the task. From these 15 journals, all the abstracts from years 1998 to 2008 which include one of the following eight chemicals were included: 1,3-Butadiene, Benzo(a)pyrene, Chloroform, Diethylnitrosamine, Diethylstilbestrol, Fumonisin B1, Phenobarbital, and Styrene. These chemicals were selected by CRA experts on the basis that they (i) are well-researched using a range of scientific tests (human, animal and cellular) and (ii) represent the two most frequent Mode of Action types (MOAs): *genotoxic *and *non-genotoxic*. A MOA is an important concept in CRA: it determines the key events leading to cancer formation. Chemicals acting by a genotoxic MOA induce cancer by interacting with DNA, while chemicals acting by a nongenotoxic MOA induce cancer without interfering directly with DNA (see [29] for details). We selected 1000 abstracts (in random) from this corpus for our work: 7,985 sentences and 225,785 words in total.

### Annotation of abstracts

#### Annotation guidelines

We annotated the 1000-abstract version of the CRA corpus according to each of the schemes. We used the annotation guidelines of Liakata for S3 [unpublished data by Maria Liakata] and developed the guidelines for S1 and S2 ourselves. The new guidelines were developed via trial annotations and discussions. They provide a generic description of each scheme and its purpose, define the unit of annotation (a sentence), introduce all the scheme categories, provide advice for conflict resolution (e.g. which categories to prefer when two or several are possible within the same sentence), and include examples of annotated abstracts. Each guideline is 15 pages long. We have made them available at http://www.cl.cam.ac.uk/~yg244/10crab.html.

#### Annotation tool

**Figure 1:**
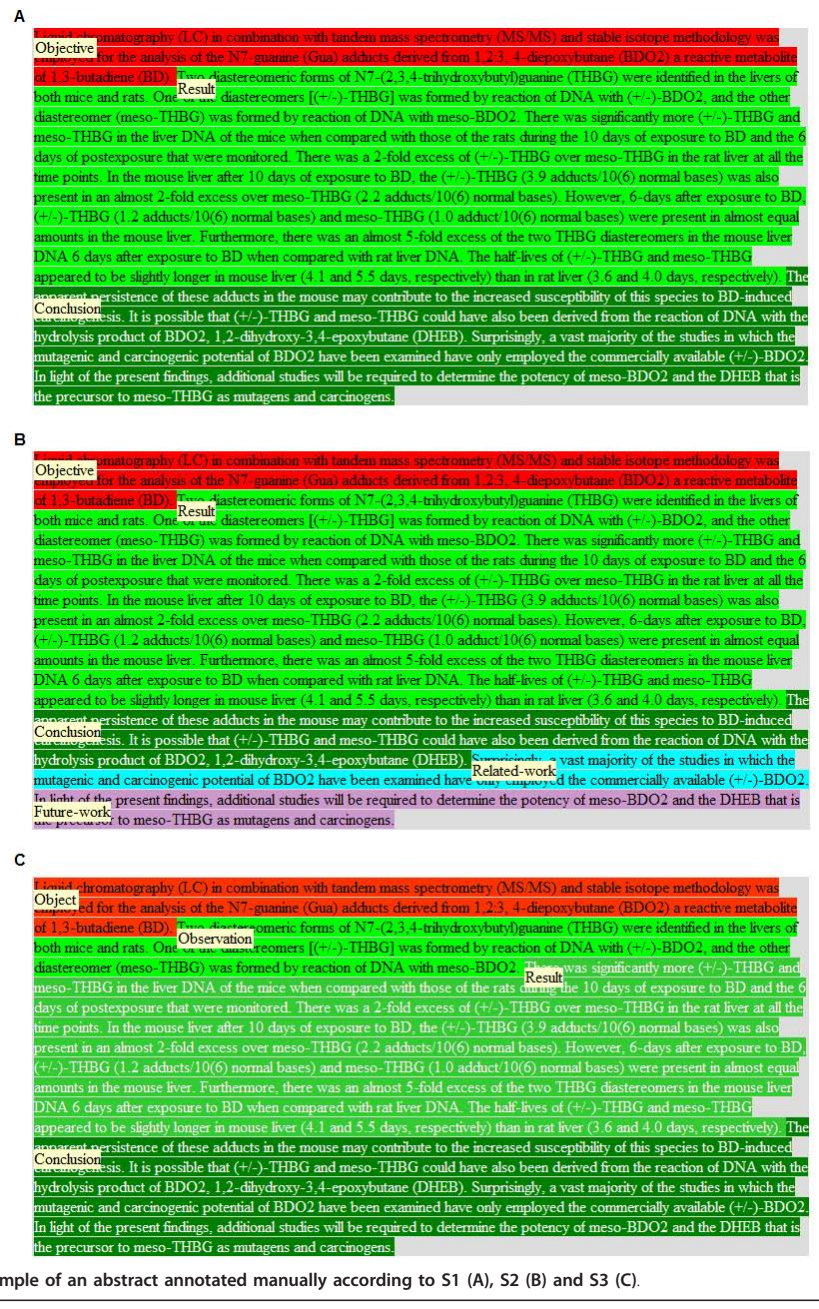
**An example of an abstract annotated manually according to S1 (A), S2 (B) and S3 (C)**.

We used the annotation tool of Korhonen et al. [29] for corpus annotation. This tool was originally developed for the annotation CRA abstracts according to the scientific evidence they contain. We modified it so that it could be used to annotate abstracts according to our schemes. It works as a Firefox plug-in. Using this tool, experts can open each MEDLINE abstract assigned to them, assign a scheme category to each sentence by highlighting it and selecting the appropriate category from a menu with a single mouse click. Highlighted sentences are displayed using colors which correspond to the different scheme categories as defined in the annotation guidelines. A screen-shot illustrating the annotation tool is provided in Figure 1. The figure shows an example abstract annotated according to each of the three schemes.

#### Description of annotation

Using the guidelines and the tool, the CRA corpus was annotated according to each of the schemes. Previous related annotation efforts have varied in terms of the expertise required from annotators. For example, Mizuta et al. [19] used a single annotator to annotate full biology articles according to S2. This person was a PhD level linguist with no training in biology. In contrast, Liakata et al. [25] used domain experts (mainly PhD students in chemistry) to annotate chemistry papers according to S3. Teufel et al. [22], in turn, used a mixed group of three annotators to annotate chemistry papers according to their recent refined AZ scheme: a PhD level computational linguist, a chemist, and a computational linguist with some experience in chemistry.

We used a single annotator (a PhD student in computational linguistics with no training in biomedical sciences) to annotate the whole CRA corpus. However, following Teufel et al., we measured inter-annotator agreement between annotators who have different expertises: the computational linguist, one domain expert (a PhD level toxicologist who is also a CRA expert) and one PhD level linguist with no training in biomedicine. The inter-annotator agreement was measured on a subset of the corpus as described later in Results section.

The annotation proceeded scheme by scheme, independently, so that annotations of one scheme were not based on any of the other two. The annotation started from the coarse-grained S1, then proceeding to S2 and finally to the finest-grained S3. The inter-annotator agreement was measured using Cohen's Kappa [32], which is the portion of agreement (P (*a*)) corrected for chance (*P*(*e*)): .

#### Comparison of annotations

The three schemes we investigate were developed independently and have separate guidelines. Thus, even though they seem to have some categories in common (e.g. METH, RES, CON) this does not necessarily guarantee that the categories cover the same information across all three schemes. We therefore investigated the relation between the schemes and the degree of overlap or complementarity between them.

We created contingency tables and calculated the chi-squared Pearson statistic, the chi-squared likelihood ratio, the contingency coefficient and Cramer's V for pairwise comparison of schemes. However, since none of these measures give an indication of the differential association between schemes (i.e. whether it goes both directions and to what extent) we also calculated the Goodman-Kruskal lambda L statistic [33]. This gives us the reduction in error for predicting the categories of one annotation scheme, if we know the categories assigned according to the other.

In addition we examined the correspondence between the actual categories of the three schemes using the paradigm of Kang et al. [34]. Kang et al. discuss a framework for subsumption checking between classes in different ontologies. They argue that if for the set of mutual instances between two classes, instances of one consistently belong to the other, we can assume that a subsumption relation holds. They suggest setting a fault tolerance threshold to cater for erroneous annotations:

where *T_k _*is the threshold, *w*(*a_i_*), *w*(*b_j_*) are the weights for instances and *X*, *Y *are the classes. We set *w*(*a_i_*) = *w*(*b_j_*) = 1, and set *T_k _*= 0.1 as a reasonable threshold, allowing at most 10% of instances to be allocated to other categories for the subsumption to hold.

### Automatic identification of information structure

Use of information structure in real-life biomedical applications requires a method capable of automatically assigning sentences in documents to appropriate scheme categories. To find out whether our schemes are machine learnable in the CRA abstract corpus, we conducted a series of classification experiments. These experiments involved extracting a range of linguistic features from each sentence in our corpus and given these features and the scheme labels in the annotated corpus, using supervised machine learning to automatically assign each sentence to the most likely category (e.g. BKG, METH, RES) of the scheme in question. Previous works in this research area have used standard text classification features (ranging from bags of words to more sophisticated features such as grammatical relations in sentences) and various well-known classifiers such as Naive Bayes [16], Support Vector Machines [26], Maximum Entropy [35], Hidden Markov Models [20] and Conditional Random Fields [21]. We used for our experiment mainly features and classifiers which have proved successful in previous works. These will be described in detail in the subsequent sections.

#### Features

The first step in automatic classification is to select features for classification. We chose a number of general purpose features suitable for all the three schemes. With the exception of our novel verb class feature, these features are similar to those employed in related works, e.g. [16,20,21,26,35]:

• **History**. There are typical patterns in the information structure so that certain categories tend to appear before others. For example, RES tends to be followed by CON rather than by BKG. Therefore, we used the category assigned to the previous sentence as a feature.

• **Location**. Categories tend to appear in typical positions in a document, e.g. BKG occurs often in the beginning and CON at the end of the abstract. We divided each abstract into ten equal parts (1-10), measured by the number of words, and defined the location (of a sentence) feature by the parts where the sentence begins and ends.

• **Word**. Like many text classification tasks, we employed all the words in the corpus as features.

• **Bi-gram**. We considered each bi-gram (combination of two adjacent word features) as a feature.

• **Verb**. Verbs are central to the meaning of sentences, and can vary from one category to another. For example, *experiment *is frequent in METH and *conclude *in CON. Previous works have used the matrix verb of each sentence as a feature. Because the matrix verb is not the only meaningful verb, we used all the verbs instead.

• **Verb Class**. Because individual verbs can result in sparse data problems, we also experimented with a novel feature: a lexical-semantic verb class (e.g. the class of EXPERIMENT verbs for verbs such as *measure *and *inject*). We obtained 60 classes by clustering verbs appearing in full cancer risk assessment articles using the approach of Sun and Korhonen [36].

• **Part-of-Speech - POS**. Tense tends to vary from one category to another, e.g. past is common in RES and past participle in CON. We used the part-of-speech (POS) tag of each verb assigned by the C&C tagger [37] as a feature.

• **Grammatical Relation - GR**. Structural information about heads and dependents has proved useful in text classification. We used grammatical relations (GRs) returned by a parser as features. They consist of a named relation, a head and a dependent, and possibly extra parameters depending on the relation involved, e.g. (*dobj investigate mouse*). We created features for each subject (*ncsubj*), direct object (*dobj*), indirect object (*iobj*) and second object (*obj2*) relation in the corpus.

• **Subj and Obj**. As some GR features may suffer from data sparsity, we collected all the subjects and objects (appearing with any verbs) from GRs and used them as features. The value of such a subject (or object) feature equals 1 if it occurs in a particular sentence (and 0 if it does not occur in the sentence).

• **Voice**. There may be a correspondence between the active and passive voice and categories (e.g. passive is frequent in METH). We therefore used voice as a feature.

#### Pre-processing and feature extraction

We developed a tokenizer to detect the boundaries of sentences and to perform basic tokenization, such as separating punctuation from adjacent words e.g. in tricky biomedical terms such as *2-amino-3,8-diethylimidazo[4,5-f]quinoxaline*. We used the C&C tools [37] adapted to biomedical literature for POS tagging, lemmatization and parsing. The lemma output was used for extracting *Word, Bi-gram *and *Verb *features. The parser produced GRs for each sentence from which we extracted the *GR, Subj, Obj *and *Voice *features. We only considered the GRs relating to verbs. The "obj" marker in a subject relation indicates a verb in passive voice (e.g. *(ncsubj observed_14 differenc_5 obj)*). To control the number of features we removed the words and GRs with fewer than 2 occurrences and bi-grams with fewer than 5 occurrences, and lemmatized the lexical items for all the features.

#### Classifiers

We used Naive Bayes (NB), Support Vector Machines (SVM), and Conditional Random Fields (CRF) for classification. These methods have been used to discover information structure in previous related works, e.g. [16,21,26]. NB is a simple and fast method, and SVM and CRF have been used successfully in a wide range of text classification tasks.

NB applies Bayes' rule and Maximum Likelihood Estimation with strong independence assumptions. It aims to select the class *c *with maximum probability given the feature set *F*:

arg max*_c _P*(*c*|*F*)

SVM constructs hyperplanes in a multidimensional space that separates data points of different classes. Good separation is achieved by the hyperplane that has the largest distance from the nearest data points of any class. The hyperplane has the form *w · x - b *= 0, where *w *is the normal vector to the hyperplane. We want to maximize the distance from the hyperplane to the data points, or the distance between two parallel hyperplanes each of which separates the data. The parallel hyperplanes can be written as: *w · x - b *= 1 and *w *· *x - b *= 1, and the distance between the two is . The problem reduces to:

Minimize |*w*|

Subject to *w *· *x_i _*- *b *≥ 1 for *x_i _*of one class,

and w · *x_i _*- *b *≤ -1 for *x_i _*of the other.

CRF is an undirected graphical model which defines a distribution over the hidden states (e.g. label sequences) given the observations. The probability of a label sequence *y *given an observation sequence *x *can be written as:

where *F_j_*(*y*, *x*, *i*) is a real-valued feature function of the states, observations, and the position in the sequence; *λ_j _*is the weight of *F_j_*, and *Z*(*x*) is a normalization factor. The *λ *parameters can be learned using the LBFGS algorithm, and arg max*_y _p*(*y*|*x*) can be inferred using the Viterbi algorithm.

We used Weka [38] (employing its NB and SVM linear kernel) and CRF_++ _[39] for the classification.

#### Evaluation methods

The results were measured in terms of accuracy (*acc*), precision (*p*, degree of correctness), recall (*r*, degree of completeness), and F-Measure (*f*, harmonic mean of *p *and *r*):

We used 10-fold cross validation to avoid the possible bias introduced by relying on any one particular split of the data. The data were randomly divided into ten parts of approximately the same size. Each individual part was retained as test data and the remaining nine parts were used as training data. The process was repeated ten times with each part used once as the test data. The resulting ten estimates were then combined to give a final score. We compare our classifiers against a baseline method based on random sampling of category labels from training data and their assignment to sentences on the basis of their observed distribution.

### User test in the context of Cancer Risk Assessment

We developed a user test so that we could evaluate and compare the practical usefulness of information structure schemes for CRA.

Two schemes were selected for this test: the coarse-grained S1 and the fine-grained S3. S2 was excluded because it proved fairly similar to S1 in terms of its performance in machine learning experiments (e.g. the number and type of categories which were actually identified in abstracts; see the Results section for details).

The user test was designed independently from the schemes. The idea was to ask cancer risk assessors to look for the information they typically look for in biomedical abstracts during an early stage of their work (when seeking to obtain an overview of the scientific data available on a chemical in question). The test was designed to compare the time it takes for risk assessors to find relevant information in (i) unannotated abstracts and (ii) abstracts annotated according to the schemes. Longer reading times have been shown to indicate greater cognitive load during language comprehension [40]. Minimizing the reading time is desirable as it can help to reduce the high cost of manual CRA. Intuitively, when risk assessors look for information about e.g. the methods used in a study, they should find this information faster when pointed to those sentences which discuss methods according to our schemes. However, whether this really helps to a significant degree (in particular when using automatically annotated abstracts), was an open question - along with which scheme (the coarse or fine-grained) might be more useful for the task.

As a starting point, cancer risk assessors working in Karolinska Institutet (Stockholm, Sweden) provided us with a list of questions they consider when studying abstracts for CRA. As the questions were of varying style and granularity and focused on various parts of abstracts, they seemed ideal for the evaluation of the schemes. The majority were adopted for the user test; however, some of the open-ended questions requiring text-based inference and more elaborate answers (e.g. The endpoints of the study?) were simplified to merely test whether and how fast the information in question could be found (e.g. Are endpoints mentioned?). This yielded a more controlled experiment which was better suited for comparing the performance of different users. We ended up with the following questionnaire containing seven questions where each question has either a verbal, 'yes' or 'no', or multiple choice answer:

Q1 **What was the aim(s) of the study?**

Verbal answer

Q2 **What was the main type of the study?**

Four possible answers from which users have to select one: an animal study, human study, *In Vitro *study or a combined study. Depending on the answer selected, three follow-up questions apply which each require a 'yes' or 'no' answer. For example, the following questions apply to a human study:

Q3a **Is exposure length mentioned?**

Q3b **Is group size mentioned?**

Q3c **Are endpoints mentioned?**

Q4 **Positive Results?**

Three possible answers: 'yes', 'no' or 'unclear'

Q5 **Author's conclusions?**

Verbal answer

We designed an on-line form which shows an abstract (and the name of the chemical which the abstract focuses on) on the top of the page and each question on the bottom of the page. The questions are displayed to experts one at a time, in the sequential order shown above. The idea of the test is to record the time it takes for an expert to answer each question. This is done by asking them to press 'start', 'next' and 'complete' buttons during different phases of the test, as appropriate.

**Figure 2:**
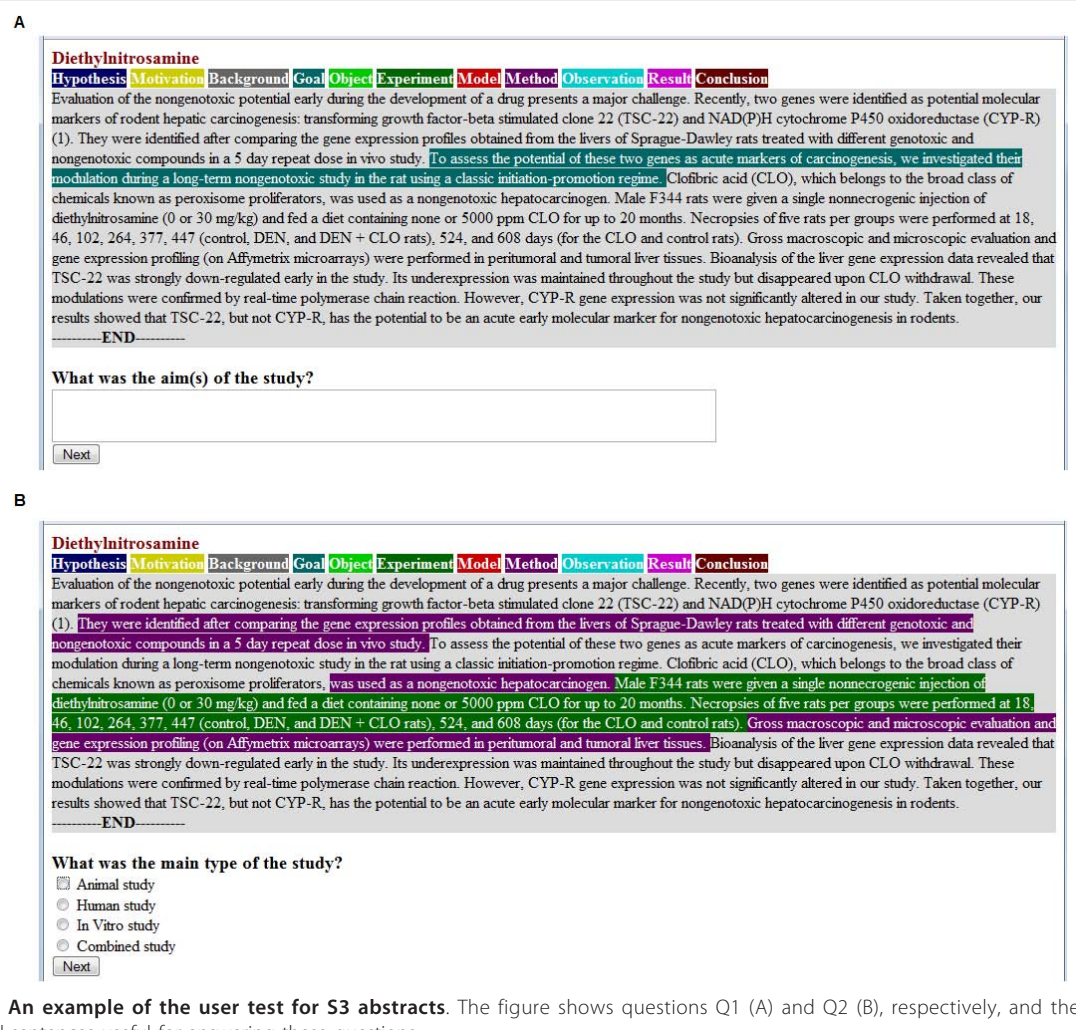
**An example of the user test for S3 abstracts**. The figure shows questions Q1 (A) and Q2 (B), respectively, and the scheme-annotated sentences useful for answering these questions.

Two screen-shots illustrating the test are shown in Figure 2. They show the same abstract annotated according to S3 for questions 1 and 2, respectively. As illustrated in the screen-shorts, although the whole abstract is shown to experts with each question, only 1-2 scheme categories are highlighted (with colors) per question, as to attract experts' attention. Those are the scheme categories which are most likely to contain an answer to the particular question. We settled for this option after conducting a pilot study which showed that users found abstracts annotated according to all the (or all the potentially relevant) scheme categories confusing rather than helpful.

Highlighting only the 1-2 most relevant categories required creating a mapping between the questions and the potentially relevant scheme categories and investigating which of the categories are really the most important ones for answering each question. We asked an expert (one of the risk assessors working at Karolinska Institutet) to examine 30 abstracts which had been manually annotated for S1 and S3 and to indicate, for each question, all the possible categories where an answer to the question could be found. This pilot study showed that although it was often possible to find an answer in several categories, there were 1-2 dominant categories which nearly always included the answer. For S1, a single dominant category could be identified for each of the questions. For S3, a single category was found for five of the questions, and two questions had two equally dominating categories which were both included because they were usually mutually exclusive. Table 2 shows all the possible categories per question, and the 1-2 dominant ones per scheme which we used in our test.

**Table 2:** The mapping between the questions in the CRA questionnaire and scheme categories

	S1		S3	
	Possible	Dominant	Possible	Dominant
Q1	OBJ	OBJ	GOAL, OBJT	GOAL, OBJ
Q2	METH, RES	METH	METH, EXP, MOD, OBS, RES	METH, EXP
Q3A	METH, RES	METH	METH, EXP, MOD, OBS, RES	EXP
Q3B	METH, RES	METH	METH, EXP, MOD, OBS, RES	EXP
Q3C	METH, RES	RES	METH, EXP, MOD, OBS, RES	OBS
Q4	RES, CON	RES	OBS, RES, CON	OBS
Q5	CON	CON	CON	CON

(i) shows all the possible categories and (ii) shows the dominant categories. The latter were used in the user test.

Three experts participated in our test: two professor level experts with a long experience in CRA (over 25 years each) - **A **and **B **- and one more junior expert: **C **who has a PhD in toxicology and over 5 years of experience in CRA. We selected 120 abstracts from the CRA corpus for this test, in random but subject to the constraint that they were similar in length and focused on one of the four chemicals: butadiene, diethylnitrosamine, diethylstilbestrol, and phenobarbital. Each expert was presented with the same set of 120 abstracts. The abstracts were divided into 5 groups (each including around 24 abstracts) so that each expert was presented with:

**S0**: unannotated abstracts,

**S1**: abstracts annotated manually according to S1,

**S3**: abstracts annotated manually according to S3,

**S1'**: abstracts automatically annotated according to S1 using the SVM classifier, and

**S3'**: abstracts automatically annotated according to S3 using the SVM classifier.

The results were measured in terms of the (i) total time it took for the experts to examine each abstract in the five groups above, and (ii) the percentage of time each expert saved when examining scheme annotated abstracts vs. unannotated ones. We also measured the statistical significance of the differences using the Mann-Whitney U Test [41,42]. The results were measured in p-value, and the chosen significance level was 0.05. Finally, we examined whether automatic annotations affected the quality of the expert answers. We did this by comparing the agreement in expert answers between S0, S1' and S3' annotated abstracts.

## Results

### The annotated corpus and inter-annotator agreement

The corpus annotation work took 45, 50 and 90 hours in total for S1, S2 and S3, respectively. Table 3 shows the distribution of sentences per scheme category in the resulting corpus. We see that for S1, all the four categories appear in abstracts with sufficient frequency, with RES being the most frequent category (accounting for 40% of the corpus). For S2, RES is also the most frequent category (again accounting for 40% of the corpus). Four other S2 categories appear in the corpus data with reasonable frequency: BKG, OBJ, METH and CON, which cover 8-18% of the corpus each. Two categories are very low in frequency, only covering 1% of the corpus each: REL and FUT. Also for S3, RES is the most frequent category (accounting for 32% of the corpus). For S3, six other categories cover 6-14% of the corpus each (BKG, OBJT, EXP, METH, OBS and CON), while four categories cover 1-4% (HYP, MOT, GOAL, and MOD). All the scheme categories we set to explore thus did appear in abstracts, but some categories belonging to the schemes that have been developed for full papers are rare. However, some of these categories have proven infrequent also in full papers [25,26].

**Table 3:** The distribution of words and sentences in the scheme-annotated CRA corpus

S1	OBJ	METH	RES	CON								
	61483	39163	89575	35564	Words							
	2145	1396	3203	1241	Sentences							
	27%	17%	40%	16%	Sentences							
**S2**	BKG	OBJ	METH	RES	CON	REL	FUT					
	36828	23493	41544	89538	30752	2456	1174	Words				
	1429	674	1473	3185	1082	95	47	Sentences				
	18%	8%	18%	40%	14%	1%	1%	Sentences				

**S3**	HYP	MOT	BKG	GOAL	OBJT	EXP	MOD	METH	OBS	RES	CON	
	2676	4277	28028	10612	15894	22444	1157	17982	17402	75951	29362	Words
	99	172	1088	294	474	805	41	637	744	2582	1049	Sentences
	1%	2%	14%	4%	6%	10%	1%	8%	9%	32%	13%	Sentences

We measured the inter-annotator agreement on 300 abstracts (i.e. a third of the corpus) using three annotators: one linguist, one expert in CRA, and the computational linguist who annotated all the corpus. We calculated Cohen's kappa [32] between each pair of annotators and averaged the results. The inter-annotator agreement was *κ *= 0.84, *κ *= 0.85, and *κ *= 0.50 for S1, S2, and S3, respectively. According to [43], the agreement 0.81-1.00 is perfect and 0.41-0.60 is moderate. S1 and S2 are thus the easiest schemes for the annotators and S3 the most challenging. This is not surprising as S3 is the scheme with the finest granularity. Its reliable identification may require a longer period of training and possibly improved guidelines. Moreover, previous annotation efforts using S3 have used domain experts for annotation [25,31]. For S3 the best agreement was between the domain expert and the linguist (*κ *= 0.60). For S1 and S2 the best agreement was between the linguist and the computational linguist (*κ *= 0.87 and *κ *= 0.88, respectively).

Table 4, 5, and 6 present a confusion matrix for S1, S2 and S3, respectively. A confusion matrix shows the categories the domain expert (**E**) and the linguist (**L**) (dis)agreed on. For S1, we can see that the annotators had trouble distinguishing between OBJ and METH, and RES and CON. For instance, there are 88 sentences labeled with METH by the domain expert and with OBJ by the linguist. Also there are 158 sentences labeled with CON by the expert and with RES by the linguist. Similar confusions can be observed between OBJ and METH, and RES and CON for S2, and between GOAL and OBJT, EXP and METH, OBS and RES, and RES and CON for S3. These problems may arise from the sentences that have two (or more) parts representing different categories (e.g. RES and CON in a single sentence). In addition to improving guidelines on these cases and providing annotators with longer training, one possible solution would be to improve the annotation strategy, for example, to use a smaller unit of annotation than a sentence, e.g. a clause.

**Table 4:** Confusion matrix for inter-annotator agreement on the CRA corpus: linguist (L) vs. domain expert (E) - S1

OBJ	METH	RES	CON	L/E
**632**	29	9	4	OBJ

88	**429**	19	1	METH

5	17	**913**	18	RES

11	4	158	**403**	CON

**Table 5:** Confusion matrix for inter-annotator agreement on the CRA corpus: linguist (L) vs. domain expert (E) - S2

BKG	OBJ	METH	RES	CON	REL	FUT	L/E
**438**	0	2	1	3	2	0	BKG

22	**211**	53	7	2	0	0	OBJ

5	17	**431**	16	0	1	0	METH

9	4	18	**935**	17	2	0	RES

7	1	5	131	**337**	4	7	CON

2	0	0	11	13	**24**	0	REL

0	0	0	0	2	0	**9**	FUT

**Table 6:** Confusion matrix for inter-annotator agreement on the CRA corpus: linguist (L) vs. domain expert (E) - S3

HYP	MOT	BKG	GOAL	OBJT	EXP	MOD	METH	OBS	RES	CON	L/E
**16**	0	12	1	0	0	0	0	0	0	5	HYP

5	**48**	13	0	2	0	0	4	0	1	0	MOT

11	6	**316**	0	3	0	0	4	1	1	2	BKG

2	4	4	**87**	80	7	0	10	0	0	0	GOAL

0	0	0	2	**39**	3	0	9	0	0	0	OBJT

0	0	1	1	7	**190**	0	66	5	8	0	EXP

0	0	3	1	4	7	**5**	13	0	2	1	MOD

0	0	8	7	25	63	5	**92**	3	10	1	METH

0	0	4	0	1	3	0	9	**183**	285	1	OBS

0	0	3	0	0	2	3	8	53	**466**	10	RES

0	1	5	1	1	0	1	3	9	105	**337**	CON

### Comparison of the schemes in terms of annotations

We used the resulting annotations to compare the degree of overlap between the schemes. Table 7 shows the results of our pairwise comparison. The chi-squared Pearson statistic, the chi-squared likelihood ratio, the contingency coefficient and Cramer's V each show a definite correlation between the rows and columns for the three schemes. When calculating the Goodman-Kruskal lambda L statistic [33], using the categories of S1 as the independent variables, we obtained a lambda of over 0.72 which suggests a 72% reduction in error in predicting S2 categories and 47% reduction in error in predicting S3 categories. With S2 categories being the independent variables, we obtain a reduction in error of 88% when predicting S1 and 55% when predicting S3 categories. The lower lambdas for predicting S3 are hardly surprising as S3 has 11 categories as opposed to 4 and 7 for S1 and S2 respectively. S3 on the other hand has strong predictive power in predicting the categories of S1 and S2 with lambdas of 0.86 and 0.84 respectively. In terms of association, S1 and S2 seem to be thus more strongly associated, followed by S1 and S3 and then S2 and S3.

**Table 7:** Association measures between schemes S1, S2, S3

	S1 vs S2			S1 vs S3			S2 vs S3		
	***X*^2^**	**df**	***P***	***X*^2^**	**df**	***P***	***X*^2^**	**df**	***P***

**Likelihood Ratio**	5577.1	18	0	5363.6	30	0	6293.4	60	0
**Pearson**	6613.0	18	0	6371.0	30	0	8554.7	60	0

**Contingency Coeff**	0.842			0.837			0.871		
**Cramer's V**	0.901			0.885			0.725		

**Figure 3:**
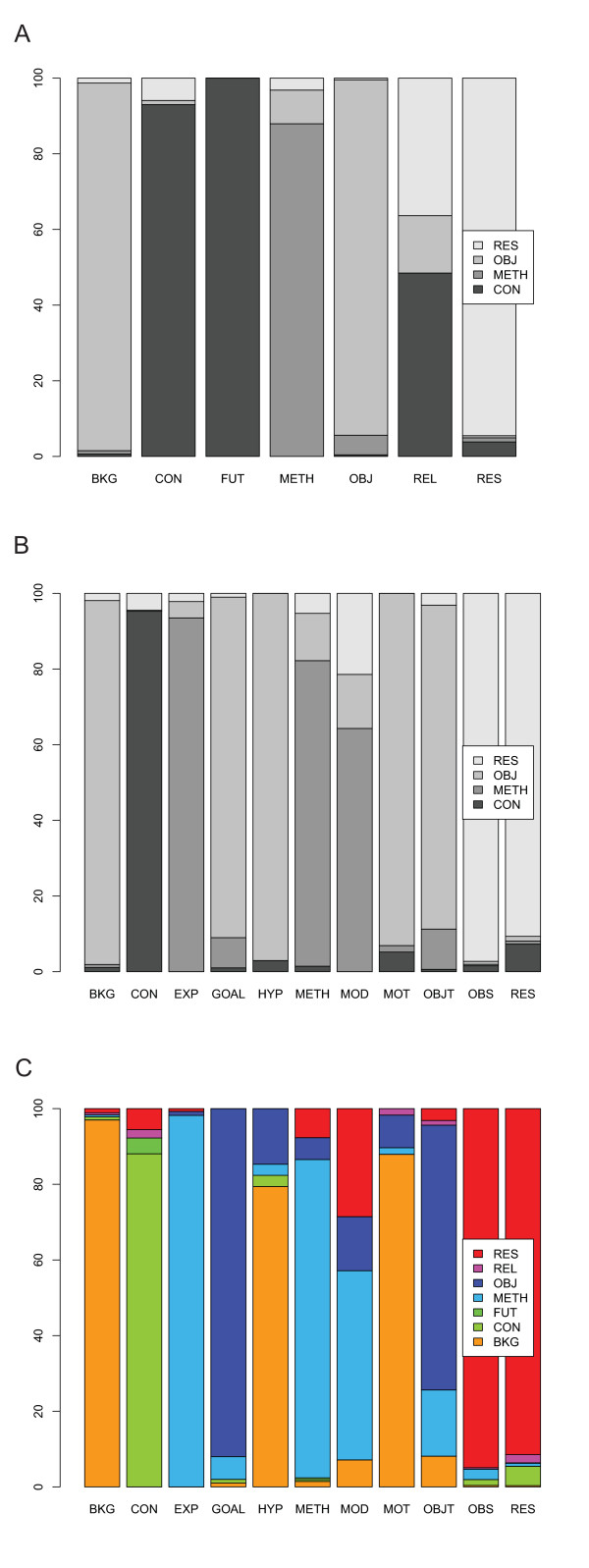
**Comparison of the three schemes in terms of manual annotations**. The figure shows pairwise interpretation of categories of one scheme in terms of the categories of the other: S2 to S1 mapping in A, S3 to S1 mapping in B and S3 to S2 mapping in C.

The correspondence between the actual categories of the three schemes is visualized in Figure 3. Take S3 BKG and S1 OBJ for example. The former maps to the latter for 96% of cases, whereas the latter maps to a number of categories in S3, namely 49% BKG, 19% GOAL, 11% MOT and 19% OBJT. It therefore would seem that S3 BKG is subsumed by S1 OBJ but not the other way round. According to the subsumption checking approach of [34], if we take *X *to be S3 BKG and *Y *to be S1 OBJ, we get 0.039 < 0.1; therefore the subsumption relation S3 BKG ⊆ S1 OBJ holds.

Take, for another example, S3 BKG and S2 BKG. The former maps to the latter in 97% of cases, whereas the latter maps to 78% BKG, 11.6% HYP, 11% MOT, 9% METH in S3. The subsumption relation is one-way: S3 BKG ⊆ S2 OBJ (with 0.03 < 0.1). Similarly, S2 BKG maps to S1 OBJ in 97% of cases, whereas S1 OBJ maps to 61.4% BKG, 30% OBJ and 9% METH in S2. The subsumption relation S2 BKG ⊆ S1 OBJ holds (with 0.029 < 0.1). Therefore, we have a subsumption relation of the type: S3 BKG ⊆ S2 BKG ⊆ S1 OBJ.

We follow the same procedure for the rest of the categories. The subsumption relations between scheme categories are summarized below:

S3 HYP ⊆ (S2 BKG ∪ S2 OBJ) S1 OBJ

S3 MOT ⊆ (S2 BKG ∪ S2 OBJ) ⊆ S1 OBJ

S3 BKG ⊆ S2 BKG ⊆ S1 OBJ

S3 GOAL ⊆ S2 OBJ S1 ⊆ OBJ

S3 OBJT ⊆ (S2 OBJ ∪ S2 METH ∪ S2 BKG) ⊆ (S1 OBJ ∪ S1 METH)

S3 EXP ⊆ S1 METH ⊆ S2 METH

S3 MOD ⊆ (S2 METH ∪ S2 OBJ ∪ S2 RES) ⊆ (S1 METH ∪ S1 OBJ ∪ S1 RES)

S3 METH ⊆ (S2 METH ∪ S2 OBJ ∪ S2 RES) ⊆ (S1 METH ∪ S1 OBJ ∪ S1 RES)

S3 OBS ⊆ S2 RES ≡ S1 RES

S3 RES ⊆ S2 RES ≡ S1 RES

S3 CON ⊆ (S2 CON ∪ S2 RES ∪ S2 FUT ∪ S2 REL) ⊆ (S1 CON ∪ S1 RES)

Based on the above analysis, it is clear that all categories in S3 are subsumed by categories in S2 which are in turn subsumed or equivalent to categories in S1. It is therefore reasonable to assume a subsumption relation between the three schemes of the type S3 ⊆ S2 ⊆ S1. This also agrees with the values of the Kruskall-lambda statistic above, according to which if we know S3 categories the likelihood of predicting S2 and S1 categories is high (84% and 86% reduction in error respectively) and decreases if we try to predict S3 when knowing S2 (55% error reduction) or S1 (47% error reduction).

This subsumption relation is an interesting outcome given that the three different schemes have such different origins.

### Automatic classification

Table 8 shows F-measure results when using each individual feature alone, and Table 9 when using all the features but the individual feature in question. In these two tables, we only report the results for SVM which performed better than other methods. Although we have results for most scheme categories, the results for some are missing due to the lack of sufficient training data (see Table 3), or due to a small feature set (e.g. *History *alone).

**Table 8:** F-Measure results when using each individual feature alone

		a	b	c	d	e	f	g	h	i	j	k
**S1**	OBJ	.39	.83	.71	.69	.52	.45	.45	.45	.54	.39	-
	METH	-	.47	.81	.74	.63	.49	-	.46	.03	.42	.51
	RES	-	.76	.85	.86	.76	.70	.72	.69	.70	.68	.54
	CON	-	.72	.70	.65	.63	.53	.49	.57	.68	.20	-

**S2**	BKG	.26	.73	.69	.67	.45	.38	.56	.33	.33	.29	-
	OBJ	-	.13	.72	.68	.54	.63	-	.49	.48	.20	-
	METH	-	.50	.81	.72	.64	.47	-	.47	.03	.42	.51
	RES	-	.76	.85	.87	.76	.72	.72	.70	.69	.68	.54
	CON	-	.70	.73	.71	.62	.51	.40	.61	.67	.23	-
	REL	-	-	-	-	-	-	-	-	-	-	-
	FUT	-	-	-	-	-	-	-	-	-	-	-

**S3**	HYP	-	-	-	-	.67	-	-	-	-	-	-
	MOT	.18	.57	.70	.49	.39	.13	.36	.33	.30	.40	-
	BKG	-	-	.54	.40	.21	-	-	.11	.06	.06	-
	GOAL	-	-	.53	.33	.22	-	.19	.31	-	.25	-
	OBJT	-	-	.73	.63	.60	.10	-	.26	.32	-	-
	EXP	-	.22	.63	.46	.33	.30	-	.31	.07	.44	.25
	MOD	-	-	-	-	-	-	-	-	-	-	-
	METH	-	-	.82	.61	.39	.39	-	.50	-	.37	-
	OBS	-	.59	.75	.71	.63	.56	.56	.54	.48	.52	.47
	RES	-	-	.87	.73	.41	.34	-	.38	.24	.35	-
	CON	-	.74	.68	.65	.65	.50	.48	.49	.55	.21	-

**a-k**: History, Location, Word, Bi-gram, Verb, Verb Class, POS, GR, Subj, Obj, Voice

**Table 9:** F-Measure results using all the features and all but one of the features

		ALL	A	B	C	D	E	F	G	H	I	J	K
**S1**	OBJ	.90	.89	.87	.92	.90	.90	.91	.91	.91	.92	.91	.88
	METH	.80	.81	.80	.80	.79	.81	.79	.80	.80	.80	.81	.81
	RES	.88	.90	.88	.90	.88	.90	.88	.88	.88	.89	.89	.90
	CON	.86	.85	.82	.87	.88	.90	.90	.88	.89	.88	.88	.90

**S2**	BKG	.91	.94	.90	.90	.93	.94	.94	.91	.93	.94	.92	.94
	OBJ	.72	.78	.84	.78	.83	.88	.84	.81	.83	.84	.78	.83
	METH	.81	.83	.80	.81	.80	.85	.80	.78	.81	.81	.82	.83
	RES	.88	.90	.88	.89	.88	.91	.89	.89	.90	.90	.90	.89
	CON	.84	.83	.77	.83	.86	.88	.86	.87	.88	.89	.88	.81
	REL	-	-	-	-	-	-	-	-	-	-	-	-
	FUT	-	1.0	1.0	1.0	1.0	1.0	1.0	1.0	1.0	1.0	1.0	1.0

**S3**	HYP	-	-	-	-	-	-	-	-	-	-	-	-
	MOT	.82	.84	.80	.76	.82	.82	.83	.78	.83	.83	.83	.83
	BKG	.59	.60	.60	.54	.67	.62	.62	.59	.61	.61	.62	.61
	GOAL	.62	.67	.67	.62	.71	.62	.67	.43	.67	.67	.67	.62
	OBJT	.88	.85	.83	.74	.83	.85	.83	.74	.83	.83	.83	.85
	EXP	.72	.68	.72	.53	.65	.70	.72	.73	.74	.74	.72	.68
	MOD	-	-	-	-	-	-	-	-	-	-	-	-
	METH	.87	.86	.87	.66	.85	.89	.87	.88	.86	.86	.87	.86
	OBS	.82	.81	.84	.72	.80	.82	.81	.80	.82	.82	.81	.81
	RES	.87	.87	.88	.74	.87	.86	.87	.86	.87	.87	.87	.88
	CON	.88	.88	.82	.88	.83	.87	.87	.84	.87	.88	.87	.86

**A-K**: History, Location, Word, Bi-gram, Verb, Verb Class, POS, GR, Subj, Obj, Voice

We have 1.0 for FUT in S2 probably because the size of the training data is just right, and the model doesn't over-fit the data. We make this assumption because we have 1.0 for almost all the categories on the training data, but only for FUT on the test data.

Looking at individual features alone, *Word, Bi-gram *and *Verb *perform the best for all the schemes, and *History *and *Voice *perform the worst. In fact *History *performs very well on the training data, but on the test data we can only use estimates rather than the actual labels; an uncertain estimate of the feature at the beginning of the abstract will introduce further uncertainty later on, leading to poor overall results. The *Voice *feature works only for RES and METH for S1 and S2, and for OBS for S3. This feature is probably only meaningful for some of the categories. When using all but one of the features, S1 and S2 suffer the most from the absence of *Location*, while S3 from the absence of *Word/POS*. *Verb Class *on its own performs worse than *Verb*, however when combined with other features it performs better: leave-Verb-out outperforms leave-Verb Class-out.

Table 10 shows the results for the baseline (BL), and the best results for NB, SVM, and CRF. NB, SVM, and CRF perform clearly better than BL for all the schemes. SVM performs the best among all the classifiers. CRF performs fairly well on S1 and S2 but not on the most fine-grained S3. NB performs well on S1, but not equally well on S2 and S3, which have a higher number of categories, some of which are low in frequency (see Table 3).

**Table 10:** Baseline and best results for NB, SVM, CRF

		**Acc**.	F-Measure										
**S1**			OBJ	METH	RES	CON							
	BL	.29	.23	.23	.39	.18							
	NB	.82	.85	.75	.85	.71							
	SVM	.89	.90	.81	.90	.90							
	CRF	.85	.87	.72	.87	.81							

**S2**			BKG	OBJ	METH	RES	CON	REL	FUT				
	BL	.25	.13	.08	.22	.40	.13	-	-				
	NB	.76	.79	.25	.70	.83	.66	-	-				
	SVM	.90	.94	.88	.85	.91	.88	1.0	-				
	CRF	.85	.92	.69	.77	.88	.75	-	.33				

**S3**			HYP	MOT	BKG	GOAL	OBJT	EXP	MOD	METH	OBS	RES	CON
	BL	.15	-	.10	.06	.04	.06	.11	-	.13	.24	.15	.17
	NB	.53	-	.56	-	-	-	.30	-	.32	.61	.59	.62
	SVM	.81	-	.82	.62	.62	.85	.70	-	.89	.82	.86	.87
	CRF	.71	-	.74	.49	.72	.67	.59	-	.58	.71	.56	.82

For S1, SVM finds all the four scheme categories with the accuracy of 89%. F-measure is 90 for OBJ, RES and CON and 81 for METH. For S2, the classifier finds six of the seven categories, with the accuracy of 90% and the average F-measure of 91 for the six categories. As with S2, METH has the lowest performance (at 85 F-measure); the one missing category (REL) appears in our abstract data with very low frequency (see Table 3).

For S3, SVM uncovers as many as nine of the eleven categories with the accuracy of 81%. Six categories perform well, with F-measure higher than 80. EXP, BKG and GOAL have F-measure of 70, 62 and 62, respectively. Like the missing categories HYP and MOD, GOAL is very low in frequency. The lower performance of the higher frequency EXP and BKG is probably due to low precision in distinguishing between EXP and METH, and BKG and other categories, respectively.

### User test

The results of the user test are presented in Table 11 and 12. Table 11 shows the total time it took for the experts (A, B, and C) to do the user test for abstracts belonging to groups S0, S1, S3, S1' and S3', respectively (see the User Test in the Methods section for details of the experts and abstract groups), along with the percentage of time the experts saved when examining scheme annotated abstracts vs. unannotated ones. Columns 2-8 show the results for each individual question and column 9 shows the overall performance. TIME stands for the sample mean (measured in seconds), and SAVE for the percentage of the time savings. Table 12 shows, for the three experts, the statistical significance of the differences between all the eight scheme pairs (e.g. S0 vs. S1, S0 vs. S3, etc). The statistical significance is indicated using p-values of the Mann-Whitney U Test (see Methods section for details).

**Table 11:** Time measures for the user test

		Q1		Q2		Q3a		Q3b		Q3c		Q4		Q5		TOTAL	
		**TIME**	**SAVE**	**TIME**	**SAVE**	**TIME**	**SAVE**	**TIME**	**SAVE**	**TIME**	**SAVE**	**TIME**	**SAVE**	**TIME**	**SAVE**	**TIME**	**SAVE**
**S0**	A	15.3		9.4		8.7		4.4		8.9		9.4		13.3		69.5	
	B	27.1		18.8		15.5		8.5		14.8		13.5		18.0		116.1	
	C	19.3		12.0		17.9		4.9		9.6		18.4		20.9		102.9	

**S1**	A	15.3	0%	7.4	22%	6.2	28%	4.2	5%	5.8	35%	7.4	21%	11.9	11%	58.2	16%
	B	17.0	37%	7.9	58%	8.5	45%	4.8	43%	6.8	54%	9.9	27%	13.1	27%	67.9	42%
	C	15.8	18%	6.4	47%	8.8	51%	3.8	21%	5.8	40%	12.5	32%	12.5	40%	65.6	36%

**S3**	A	13.1	15%	7.9	17%	5.6	35%	3.9	11%	5.7	36%	6.4	32%	11.9	11%	54.5	22%
	B	15.9	41%	8.9	53%	7.2	53%	4.7	45%	7.8	47%	6.1	55%	12.0	33%	62.6	46%
	C	15.4	20%	5.9	51%	8.5	53%	3.8	22%	6.9	29%	11.8	36%	11.4	45%	63.7	38%

**S1'**	A	15.0	3%	9.4	1%	6.3	28%	4.2	5%	7.1	20%	7.4	22%	12.5	6%	61.8	11%
	B	18.4	32%	12.4	34%	9.4	39%	8.1	5%	8.2	44%	6.7	50%	14.2	21%	77.5	33%
	C	18.5	4%	12.3	-3%	13.9	22%	6.3	-29%	8.6	11%	12.8	31%	12.9	38%	85.3	17%

**S3'**	A	13.0	16%	8.3	12%	6.6	24%	4.9	-11%	6.5	27%	6.8	28%	11.5	14%	57.6	17%
	B	23.9	12%	14.5	23%	11.4	26%	7.8	8%	10.1	32%	7.2	47%	15.3	15%	90.2	22%
	C	17.1	11%	12.0	0%	15.1	16%	4.8	1%	8.3	14%	11.9	35%	15.8	24%	84.9	17%

The table shows the time it takes for the CRA experts (A, B and C) to answer the questions in the questionnaire (sample mean) and the percentage of time they save using scheme annotations.

**Table 12:** Significance of the results in the previous table according to the Mann-Whitney U Test (p-value)

		Q1	Q2	Q3a	Q3b	Q3c	Q4	Q5	TOTAL
**S0 vs S1**	A	.594	**.048**	.058	.247	**.000**	.081	.109	**.001**
	B	**.000**	**.037**	**.007**	**.017**	**.001**	.352	**.004**	**.000**
	C	.192	**.013**	**.001**	.076	.146	**.012**	**.001**	**.000**

**S0 vs S3**	A	**.015**	.074	**.008**	.096	**.000**	**.010**	**.033**	**.000**
	B	**.000**	.058	**.000**	**.010**	**.005**	**.027**	**.000**	**.000**
	C	**.042**	**.006**	**.000**	.369	.574	**.003**	**.000**	**.000**

**S1 vs S3**	A	**.009**	.663	.190	.743	.676	.175	.486	.152
	B	.508	.592	.174	.729	.170	**.041**	.623	.340
	C	.488	.800	.855	.338	.357	.405	.673	.420

**S1 vs S1'**	A	.443	.286	.590	.546	.294	.599	.351	.316
	B	**.037**	.201	.673	**.014**	.188	.106	.600	.058
	C	.394	**.006**	.053	**.002**	**.025**	.900	.538	**.000**

**S3 vs S3'**	A	.677	.350	.315	.094	.102	.720	.719	.458
	B	**.010**	.144	**.005**	**.002**	.356	.542	.058	**.000**
	C	.253	**.006**	**.001**	.052	.341	.579	**.006**	**.001**

**S0 vs S1'**	A	.600	.331	**.028**	.627	**.009**	.118	.709	**.044**
	B	**.001**	.382	**.017**	.894	**.010**	.066	**.023**	**.000**
	C	.576	.820	.127	.076	.668	**.024**	**.004**	**.017**

**S0 vs S3'**	A	**.003**	.285	.118	.704	**.002**	**.029**	**.015**	**.000**
	B	.232	.747	.073	.919	.107	**.050**	.144	**.006**
	C	.362	.619	.468	.252	.810	**.008**	.134	**.028**

**S1' vs S3'**	A	**.031**	.695	.589	.458	.873	.750	.107	.141
	B	.075	.600	.358	.601	.260	.873	.474	**.045**
	C	.693	.898	.291	.377	.732	.898	.127	.907

Looking at the overall performance figures, the average time spent with unannotated abstracts (S0) was 69.5 seconds for A, 116.1 for B, and 102.9 for C. All the experts spent significantly less time with scheme annotated abstracts (S1, S3, S1' and S3') than with unannotated ones (S0): the percentage of time saved ranges between 11% and 46%. Even A, who was the fastest expert with unannotated abstracts, saved 16%, 22%, 11% and 17% time with S1, S3, S1' and S3', respectively. For other users the savings in time were bigger.

As expected, the more accurate manually annotated abstracts (S1, S3) help save more time (33% on average per expert) than automatically annotated ones (S1', S3') (19.5% on average per expert). For instance, in the case of C, S1 and S3 saved 36% and 38% of time, respectively, whereas S1' and S3' saved 17%. However, automatic annotations still clearly helped experts conduct their task faster.

Looking at individual questions, for all the users, no significant difference (p > .05) was found in results between S1' and S1 for Q3a, Q4, and Q5, and between S3' and S3 for Q3c and Q4. These are the questions which map to the frequent scheme categories with high F-Measures in machine learning experiments: RES, CON for S1', and OBS for S3', as shown in Table 10. We can therefore expect future improvements in the automatic detection of lower frequency scheme categories lead to improved performance also in user tests.

Comparing the two schemes, for each user, S3 (the fine-grained CoreSC scheme) saved more time than S1 (the coarse-grained section name -based scheme) for the majority of questions: Q1, Q3a, Q3b, Q4, and Q5. Similarly, S3' saved more time than S1' for Q1, Q2, Q3b, Q3c, and Q4 for the majority of users. These include both broader questions requiring verbal answers like Q1 and Q5, and more specific questions requiring 'yes' vs. 'no' answers like Q3a-c. Although the majority of these differences between the two schemes are not statistically significant (see Table 12), the small benefit of S3 (and the fact that S1 rarely beats S3) is still a clear trend in the data, and shows also in the TOTAL results.

We finally examined whether using automatically annotated abstracts had an impact on the experts' accuracy. We took the abstracts annotated according to S1' and S3', respectively, and compared the results B and C obtained when using these abstracts against the results A obtained when using S0 annotations of the abstracts. Interestingly, when using S1' annotations, 83-85% of the answers produced by B and C agreed with the answers produced by A. When using S3' annotations, the agreement of B and C with A was 93%. This demonstrates that the use of automatic annotations does not result in a significant drop in experts' accuracy, in particular when a fine-grained scheme such as S3 is used.

## Discussion and conclusions

The results from our corpus annotation (see Table 3) show that for the coarse-grained S1, all the four categories appear frequently in biomedical abstracts. This is not surprising because S1 was actually developed for abstracts. The inter-annotator agreement on this scheme was good and all the categories were also identified by machine learning (three of them with F of 90) yielding high overall accuracy (89%).

For S2, all the seven categories appeared in the CRA corpus and six were found by the SVM classifier. Also this scheme had good inter-annotator agreement and obtained very similar accuracy in machine learning experiments than S1: 90%.

For S3, all the eleven categories appeared in the corpus and nine of them (even some of the low frequency ones) were identified using the SVM classifier with the overall accuracy of 81%. This accuracy is surprisingly good given the high number of categories, many of which were low in frequency in the CRA corpus, and considering the low inter-annotator agreement on this scheme (note, however, that we used data annotated according to a single annotator in our machine learning experiments - therefore, the inter-annotator agreement result does not provide a reliable human upper bound for our experiments).

These results show that all the three schemes are applicable to abstracts and can be identified in them automatically with relatively high accuracy. Interestingly, our analysis in section 'Comparison of the schemes in terms of annotations' demonstrates that there is a subsumption relation between the categories of the three schemes. This is surprising since the three schemes were developed based on different principles: S1 on section names, S2 on following the knowledge claims made by authors, and S3 on tracking the structure of a scientific investigation at the level of scientific concepts. Our comparison shows that the main practical difference between the schemes is that S2 and S3 provide finer-grained information about the information structure of abstracts than S1 (even with their 2-3 low frequency or missing categories).

Ultimately, an optimal scheme will depend on the level of detail required by the application at hand. Similarly, the level of accuracy required in machine learning performance may be application-dependent. To shed light on these issues, we conducted evaluation in the context of the real-life task of CRA. In this evaluation we focused on the most general S1 and the most detailed S3 schemes only.

The user test was designed independently of the two schemes. Three cancer risk assessors were presented with a questionnaire which involved looking for information (both detailed and general) relevant for CRA in different parts of biomedical abstracts. We evaluated the time it took for the experts to answer the questions when presented with plain unannotated abstracts and those annotated manually and automatically according to S1 and S3.

The results show that all the experts saved significant amounts of time when examining abstracts highlighted for the most relevant scheme categories per question. Although manually annotated abstracts are more useful (yielding overall savings of 16-46%), automatically annotated ones lead to significant savings in time as well (11-33% overall) in comparison with unannotated abstracts. Although no statistically significant differences could be observed between S1 and S3, all the experts performed faster with the majority of questions when presented with S3-labeled abstracts. Interestingly, this tendency could be observed with both manual and automatically annotated abstracts.

It is obvious, looking at the 1-2 dominant scheme categories (which we showed to the experts per question) and comparing them to the full set of possible categories in Table 2 that our CRA questionnaire would not realize the full potential of S3. However, the fact that S3 proved at least as helpful for users as S1 despite the lower machine learning performance is promising. On the other hand, it is encouraging that a scheme as simple as S1 can be used to aid a real-world task with a significant saving in users' time.

Our user test - which is, to our knowledge, the first attempt to evaluate information structure schemes directly in the context of real-life biomedical tasks - focused on one step of CRA. This step involves looking for relevant information in abstracts, mainly to determine the usefulness of abstracts for the task. Other steps of CRA, in particular those which focus on more detailed information in full biomedical journal articles, are likely to benefit from the schemes of information structure to a greater degree. We intend to explore this avenue of work in our future experiments.

For real-life tasks involving abstracts, it would be useful to further improve machine learning performance. Previous works have not evaluated S2 or S3 on biomedical abstracts. However, Hirohata et al. [21] have evaluated S1. They showed that the amount of training data used can have a big impact on the task. They used c. 50,000 MEDLINE abstracts annotated (by the authors of the abstracts) as training data for S1. When using a small set of standard text classification features and CRF for classification, they obtained 95.5% per-sentence accuracy on 1000 abstracts. However, when only 1000 abstracts were used for training the accuracy was considerably worse; their reported per-abstract accuracy dropped from 68.8% to less than 50%. This contrasts with our CRF accuracy of 85% on 1000 abstracts. Although it would be difficult to obtain similarly huge training data for S2 and S3, this result suggests that one key to improved performance is larger training data, and this is what we plan to explore especially for S1.

In addition we plan to improve our method. We showed that our schemes are partly overlapping and that similar features and methods tend to perform the best/worst for each of the schemes. It is therefore unlikely that considerable scheme specific tuning will be necessary. However, we plan to develop our features further and to make better use of the sequential nature of information structure. Although CRF proved disappointing in our experiment, it may be worth investigating it further (by e.g. using features gathered from surrounding sentences) and also comparing it (and the SVM method) against methods such as Maximum Entropy which have proved successful in recent related works [35]. The resulting models will be evaluated both directly and in the context of CRA to provide an indication of their practical usefulness for real-world tasks.

## Availability and requirements

• **Project name: **Tool for the user test

• **Project home page: **http://www.cl.cam.ac.uk/~yg244/10crab.html

• **Operating System(s): **Platform independent (tested on Windows and Linux)

• **Programming language: **XUL and Javascript, Perl and Java for classification

• **Other requirements: **Firefox 2.0 (available from http://www.mozilla.com/en-US/firefox/all-older.html)

• **License: **The application will be freely accessible for all users.

• **Any restrictions to use by non-academics: **none

## Authors' contributions

All the authors participated actively in the work reported in this paper. AK took the main responsibility of the write-up of the paper, and together with ML and US designed, supervised and coordinated the project. YG conducted the corpus annotation work with the assistance of IS, and conducted the inter-annotator agreement tests. ML did the comparison of the schemes using the annotated corpus. YG implemented and evaluated the automatic classification approach and set up and evaluated the results of the user test. The user test was carried out by US, JH, and IS. All authors have read and accepted the final manuscript.
